# Ultrasound Irradiation as a Candidate Procedure to Improve the Transdermal Drug Delivery to the Tail Edema of a Mouse Model

**DOI:** 10.3390/ijms252211883

**Published:** 2024-11-05

**Authors:** Shinji Kumegawa, Takuya Suzuki, Kota Fujimoto, Kazuhisa Uemura, Katsuro Tachibana, Gen Yamada, Shinichi Asamura

**Affiliations:** 1 Department of Plastic and Reconstructive Surgery, Graduate School of Medicine, Wakayama Medical University, Wakayama 641-8509, Japan; 2 Department of Anatomy, Faculty of Medicine, Fukuoka University, Fukuoka 814-0180, Japan

**Keywords:** ultrasound irradiation, lymphedema, transdermal administration, steroid

## Abstract

Drug therapy for secondary lymphedema has not yet been established. Conventional oral and intravenous administration is difficult to administer in sufficient doses due to adverse events. Therefore, it is necessary to develop a transdermal delivery system that can deliver high concentrations of drugs to the edema area. In this study, we examined the efficacy of transdermal drug delivery in a mouse model of tail edema using ultrasound irradiation (sonication method). Ultrasound irradiation can deliver high-molecular-weight substances subcutaneously, and the percutaneous administration of clobetasol propionate to the mouse tail edema model prevented the enlargement of lymphatic vessels with reduced tail volume. Therefore, steroid administration utilizing ultrasound irradiation is effective in decreasing tail swelling in a mouse tail edema model. Thus, ultrasound irradiation could have the potential to innovate the treatment of secondary lymphedema by directly administering the drug to the edema.

## 1. Introduction

Lymphedema is the chronic swelling of the limbs due to impaired lymphatic function. Secondary lymphedema often arises as a complication following surgery [1]. It has been generally stated that the onset of secondary lymphedema occurs approximately in 1 in 1000 people in the US [2]. Approximately 20% of post-breast-cancer surgery patients develop secondary lymphedema [3]. Such cases as lymph node dissection for cancer treatment result in the accumulation of lymphatic fluid in tissues and swellings. This condition can severely impact a patient’s quality of life, causing chronic pain, decreased mobility, and recurrent infections [1,4]. Lymphatic insufficiency is progressive. Without appropriate treatments, the disease progresses, eventually leading to fat deposits and a condition similar to lipedema [5,6]. Indocyanine green fluorescent lymphography and other lymphoimaging methods are used to diagnose lymphedema [7]. Bioelectrical impedance analysis is being utilized to evaluate treatment efficacy [8].

Despite the advancements in surgical interventions like lymphaticovenous anastomosis and lymphatic system transfer [9,10], pharmacological options remain limited [11]. Various approaches have been tried for the noninvasive treatment of secondary lymphedema, including lymphangiogenic growth factors, anti-inflammatory drugs, tacrolimus, and antifibrotic agents, but there is no established treatment at this time [12]. Current surgical methods are mostly invasive and may not be a suitable option for all patients. Therefore, the development of noninvasive or minimally invasive treatments is crucial. However, pharmacological treatments for secondary lymphedema have not yet achieved significant progress. There are almost no approved drugs for specifically targeting conditions including fat deposition and the fibrogenesis of subcutaneous tissue. Diuretics are sometimes used for initial treatment, but their long-term administration is not recommended because they cause electrolyte imbalances. The efficacy of benzopyrones and immunological therapy is not established [1,11]. Existing palliative therapies, such as the use of Chinese herbal medicine, have shown limited promise in alleviating symptoms [13]. Moreover, scientific evidence supporting their efficacy is still weak, and these treatments are not officially recommended in the consensus document of the International Society of Lymphology [1].

The lack of effective drug therapies highlights a critical gap in the management of secondary lymphedema. Developing new pharmacological approaches will provide patients with less invasive treatment options, potentially improving adherence and outcomes. This situation requires innovative research for drug delivery methods that can effectively target the affected tissues.

The cutaneal stratum corneum acts as a barrier that prevents the diffusion of applied molecules with a molecular weight greater than 600 Dalton. For drugs to be delivered passively through the skin, they should possess around such molecular weight and exhibit sufficient lipophilicity, making the transdermal delivery of macromolecules generally challenging [14].

Sonication is a method utilizing ultrasound irradiation (hereafter, the method is termed ultrasound irradiation) to deliver high-molecular-weight substances that are normally difficult to penetrate into the skin or across cell membranes [15,16,17]. There are no studies yet on the administration of drugs utilizing ultrasound irradiation for lymphedema.

This study aims to address this gap by investigating the feasibility of transdermal administration and drug delivery using a combination of our developed mouse edema model, termed Tail Edema by Silicon sheet-mediated Transparency protocol (TEST) [18], and the ultrasound irradiation method.

There are various mouse models of chronic lymphedema, but few of them exhibit sustained, stable long-term edema [19,20,21,22,23,24,25]. We examined the efficacy of transdermal drug administration using our edema model which reproduces long-term edema [18]. Ultrasound irradiation is an approach to enhance the uptake of genes or drug molecules by inducing acoustic cavitation through ultrasound irradiation [26,27]. It is an effective gene and drug delivery procedure utilizing ultrasound without significant target tissue damages such as cell death [28,29,30].

In the current study, an examination for the efficacy of transdermal fluorescent dye administration by ultrasound irradiation was first performed. As a model treatment protocol for lymphedema, the application of transdermal clobetasol propionate, one of the most potent topical steroids [31], to the target tissue was investigated. By enhancing the permeability of the skin and ensuring efficient drug delivery to the target areas, this approach could pave the way for new treatments for secondary lymphedema. This is the first study to examine the effects of drug delivery using ultrasound irradiation on a mouse tail lymphedema model.

## 2. Results

### 2.1. Transdermal Administration of Fluorescein Isothiocyanate (FITC) Dextran by Ultrasound Irradiation

**Figure 1:**
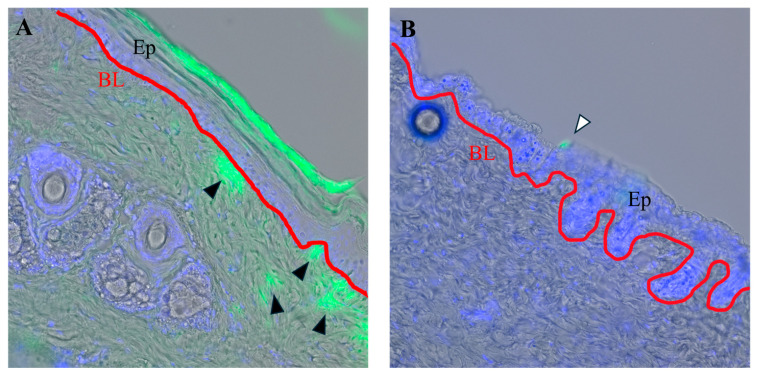
Transdermal high-molecular-weight FITC dextran delivery using ultrasound irradiation. (**A**) Fluorescence microscope image of mouse tail skin section ultrasound irradiated with FITC dextran. By this condition, 2000 kDa dextran was detected beyond the basal skin layer (black arrow heads). (**B**) Fluorescence microscope image of mouse tail skin section with FITC dextran application without ultrasound irradiation. By the condition, 2000 kDa dextran was detected in the surface of the epidermis (white arrow head) but not detected beyond the basal skin layer. Ep: epidermis, BL: basal skin layer shown by red line.

To examine the degree of transdermal high-molecular-weight substance transition by ultrasound irradiation, we first investigated whether FITC dextran could be transduced by ultrasound irradiation in a mouse tail model. The application of FITC dextran has been frequently reported for analyzing high-molecular-weight substance penetration and detection experimentally. Such molecule is also frequently utilized after various experimental treatments including electroporation [32]. A drop of 2000 kDa FITC dextran solution was applied to the skin surface of the tail of ICR mice and subsequently treated by ultrasound irradiation. The fluorescence microscopy of the corresponding frozen sections showed that FITC dextran penetrated beyond the basal skin layer into the subcutaneous tissue compared with the non-irradiated controls (Figure 1A; black arrows).

This suggests that the application of ultrasound irradiation enabled the transdermal transfusion of polymeric dextran, a high-molecular-weight marker.

### 2.2. Effect of Ultrasound-Mediated Steroid Treatments for Reducing Lymphedema Volume

To investigate the combinatorial merits of animal model utilization and drug delivery by ultrasound irradiation, the current mouse tail edema model was used to examine the effect of steroid administration to the edema area. The TEST mouse tail edema model was established recently to reproduce chronic edema formation which offers an efficient experimental model for various analyses [18].

**Figure 2:**
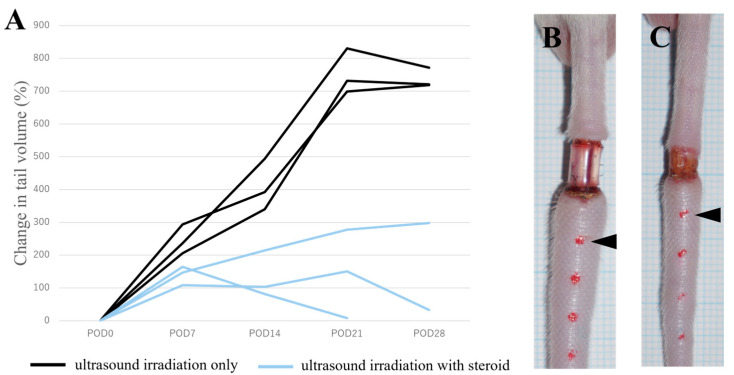
Evaluation of therapeutic effect on lymphedema by transdermal administration of clobetasol propionate using ultrasound irradiation. (**A**) The values for postoperative tail volumes are shown as previously measured [18]. This figure shows the time course values of the swelling status of lymphoedema mouse tail (at postoperative days). The ultrasound irradiation with steroid treated group (three mice, shown by blue lines) showed reduced tail volume compared with the ultrasound irradiation-only group (three mice, shown by black lines). The time point of postoperative day was indicated as POD, including POD0 as the time of the surgical treatment (0 day after surgical treatment). (**B**) Representative tail image of ultrasound irradiation-only mouse tail edema. (**C**) Representative tail image of ultrasound irradiation with steroid-treated mouse tail edema. The red dots on the tails in (**B**,**C**) represent the points for measurement. The black arrowhead indicates point C1 (red dot close to the surgical site), as previously reported [18].

In the current model, the volume of the tail lesion was calculated by the tail region encompassing 35 mm with 8 dots after four weeks after skin resection and L.V. ligation. Significantly reduced edema formation was observed in the ultrasound irradiation, with steroid groups compared with ultrasound irradiation-only controls (Figure 2).

### 2.3. Reduced Formation of Abnormally Dilated Lymphatic Vessels with Fatty Lesions

**Figure 3:**
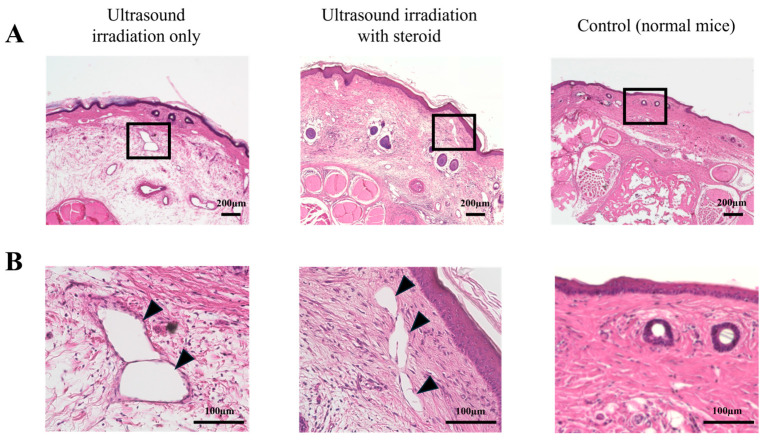
Ultrasound irradiating clobetasol propionate reduced swelled subcutaneous tissue and lymphatic vessels. (**A**) Cross-sectional histology of the mouse tail lymphedemic region with HE staining (low power field). In the ultrasound irradiation with steroid group, the thickness of dermal tissue and abnormal lymphatic vessels was reduced. The black boxes in each image indicate the magnified area in Figure 3B. (**B**) Cross-sectional histology of the mouse tail lymphedema model with HE staining (high power field). Black arrow heads indicate lymphatic vessels. Abnormally swelled lymphatic vessels were reduced in the ultrasound irradiation with steroid-treated group. Few lymphatic vessels were observed in the control group.

**Figure 4:**
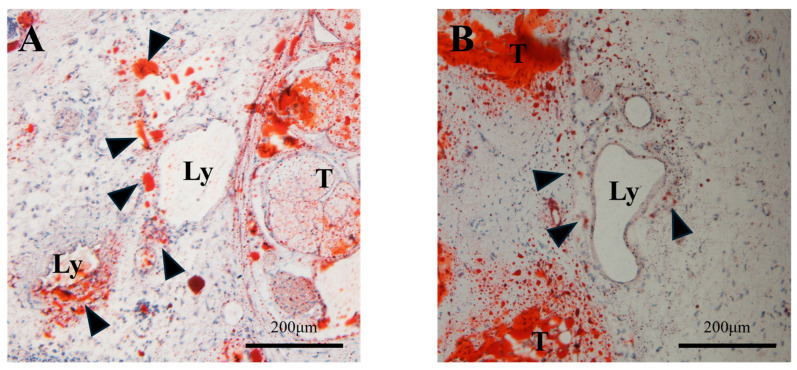
Adipose tissue region was reduced by the application of clobetasol propionate with ultrasound irradiation. Cross-sectional histology of the mouse tail lymphedema with oil-red O staining. (**A**) Black arrow heads indicate prominent adipose tissues detected around swelled lymphatic vessels of the ultrasound irradiation-only group. (**B**) Reduced formation of adipose tissues was detected in the ultrasound irradiation with steroid-treated group (indicated by black arrow heads). Ly: lymphatic vessel; T: tail tendon.

To examine the efficacy and improvement of the phenotype of ultrasound irradiation with steroid groups, various histological analyses by HE staining and oil-red O staining were performed. Lymphedema-specific features such as swelled subcutaneous tissue and enlarged L.V. were observed in the ultrasound irradiation-only groups by HE staining (Figure 3). On the other hand, such changes were rarely observed in the ultrasound irradiation with steroid group (Figure 3). Oil-red O staining was performed to detect adipose tissue and such detected region was markedly reduced in the ultrasound irradiation with steroid group (Figure 4).

### 2.4. Appearance of CD4-Positive T Cells Adjacent to the Lymphatic Vessels in the Lymphedemic Group

**Figure 5:**
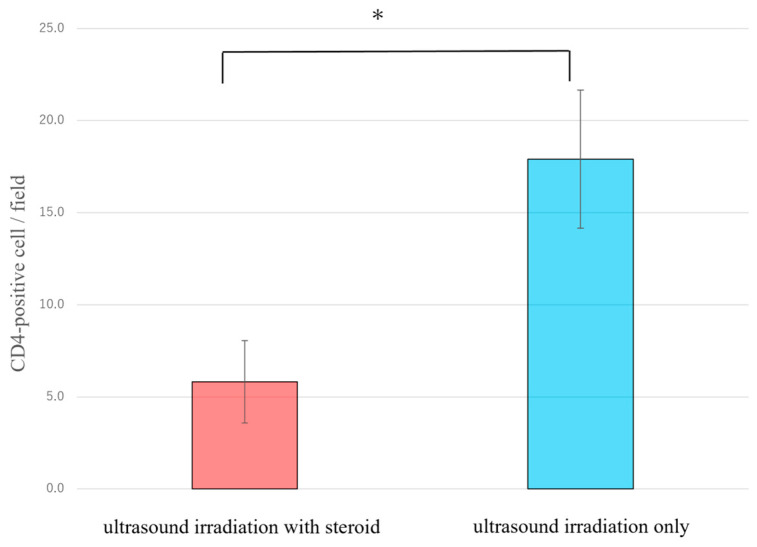
Counting the number of CD4-positive cells by tail sections showed its significant decrease in the treatment group. The vertical axis shows the number of CD4-positive cells around lymphatic vessels per field (360 μm × 270 μm). Each group contains three mice. CD4-positive cells were significantly reduced in the ultrasound irradiation with steroid-treated group (* *p* < 0.05, Student’s *t*-test).

In the early stage of lymphedema, CD4-positive T cells promote lymphogenesis through macrophage activation [21]. The appearance of CD4-positive T cells is associated with tail lymphedema formation [33]. Thus, we investigated possible changes in such a population of CD4-positive T cell counts around the L.V. Significantly reduced CD4-positive T cell counts were observed in the treated group (Figure 5).

## 3. Discussion

Here, we describe the first successful treatment of experimental tail lymphedema model by ultrasound irradiation. The current study demonstrated that ultrasound irradiation could facilitate the delivery of high-molecular-weight substances, such as FITC dextran, through the skin. These results indicate that ultrasound irradiation can effectively enhance the permeability of the skin, allowing the administration of potentially larger therapeutic molecules that would otherwise be difficult to penetrate.

### 3.1. Transdermal Administration of FITC Dextran as a Model Case

Transdermal drug delivery possesses advantages compared to other methods of administration, including less invasiveness, the avoidance of first pass effects, and the potential to deliver locally higher concentrations of drugs. However, the drugs that can be passively absorbed percutaneously are limited [14]. Consequently, several strategies have been developed to enhance transdermal drug delivery. These include ultrasound irradiation, optimizing formulations to increase skin permeability, employing drug delivery vehicles using various frequency with microneedles, and various biomolecules [14].

FITC dextran is a representative compound often used to analyze skin permeability in such transdermal drug delivery studies. Dextran is a chain polysaccharide composed of glucose, possessing a high biological safety profile. In this study, the successful transdermal administration of 2000 kDa FITC dextran in the mouse model underscores the efficiency of ultrasound irradiation in delivering relatively large molecules beyond the basal skin layer. These results suggest that ultrasound irradiation may be effective in penetrating the skin barrier in drug delivery, potentially for antibody drugs with similar molecular weights reaching 150 kDa. The ability to deliver high-molecular-weight substances to subcutaneous tissues is particularly relevant for lymphedema treatment, where the tissue delivery of therapeutic agents is necessary to alleviate symptoms effectively.

Clobetasol propionate, which was administered to the mouse tail edema model in the current study, is a substance with a molecular weight of less than 500 Da. However, it is difficult to penetrate subcutaneously enough with a short administration time (10 min), as in the current study. The results confirming the transdermal delivery of a high molecular weight marker suggest that such transdermal delivery could be possible for substances that are difficult to deliver transdermally.

Generally, shorter periods of irradiation time, such as within the range of seconds, has been often reported for ultrasound irradiation with micro- or nano-bubbles [34,35,36,37]. A limitation of the current study is the relatively long exposure time of ultrasound to the tail skin. It is generally accepted that the combinatorial injection of target molecule plus micro-bubble (or nano-bubble) is beneficial for the highly efficient transduction with a shorter time of ultrasound exposure. It is considerable that the injection of the target area enables a relatively higher exposure of the target drug and micro-bubble (or nano-bubble) inside the tissue [38,39]. However, transdermal drug delivery by topical agents is necessary for lymphedema because of the need for drug penetration into both the epidermis and subcutis. Further works are necessary to moderate the application of several possibilities.

### 3.2. Effect of Steroidal Treatment in the Tail Model of Lymphedema

The application of transdermal steroids using the mouse model and ultrasound irradiation showed promising results in reducing lymphedema volume and improving histological features associated with the condition. In lymphedema, its anti-inflammatory effects may also be useful in suppressing the symptoms. However, conventional systemic administration of steroids is difficult for the long term because of adverse events such as skin atrophy, hypopigmentation around the application site, Cushing-like syndrome, adrenal hypophysial axis suppression, osteonecrosis, and folliculitis [31]. Transdermal administration may solve these problems because it can act locally at the site of the disease.

The advantage of the tail model is that tail skin has an epidermis of similar thickness to human skin compared to the dorsal skin [40]. The penetration of macromolecules into the tail skin of mice, which have thick keratinized skin, suggests that drug administration by ultrasound irradiation might be effective also for humans.

The mechanism by which ultrasound irradiation enables the transdermal delivery of steroids is considered to involve the disordering of stratum corneum and convective flow resulting from the cavitation effects. As for the possibility of hyperthermia generated by ultrasonic irradiation, the temperature increase before and after irradiation is approximately 1.5 °C and the penetration of the drug is considered unlikely in such temperature.

In this experiment, we used clobetasol propionate as one of the potent steroids. In future studies, using moderate steroids that can be administered prophylactically or tailored to the symptoms of lymphedema should be considered. The combinatorial treatment led to significantly reduced edema formation and a decrease in the appearance of abnormally dilated L.V.s and fatty lesions. These outcomes suggest that the combined use of animal models and advanced drug delivery techniques can effectively work and treat lymphedema.

Furthermore, the histological analysis revealed the appearance of fewer CD4-positive T-cells adjacent to L.V. in the treated groups. A previous study revealed that the administration of steroids decreases T cells, especially CD4-positive T cells [41]. The current reduced CD4-positive T cell number suggests the effect of steroid local administration, which is a desirable outcome in managing lymphedema. Steroids also have an anti-inflammatory effect and could contribute to reducing lymphedema’s pathological progression. Transdermal administration by ultrasound irradiation has potential to inhibit lymphedema progression.

### 3.3. Implications for Future Research and Treatment

The results of this study may provide a foundation for further exploration into minimally invasive treatments for secondary lymphedema. The effectiveness of ultrasound irradiation in enhancing drug delivery opens new avenues for the administration of a range of therapeutic agents with relatively high molecular weights.

Moreover, the success of steroid treatment in reducing lymphedema symptoms through this method suggests that similar approaches could be adapted for other pharmacological agents. Oral Chinese herbal medicines do not directly alleviate lymphedema symptoms but contribute to the regulation of bodily fluids and exhibit mild efficacy. Additionally, oral medications such as COX inhibitors and angiogenesis-stimulating drugs have been investigated [42]. However, their systemic administration is currently limited due to the potential risk of promoting malignant tumor growth [43]. Local administration using ultrasound irradiation could lead to the development of targeted therapies that are less invasive than current surgical options and potentially more effective than existing pharmacological treatments.

## 4. Materials and Methods

### 4.1. Animals

Male ICR mice (2 months old) were purchased from CLEA Japan. All procedures and protocols have been approved by Animal Research Committee of Wakayama Medical University (approval number: 1156). We have confirmed that all methods were carried out in accordance with relevant guidelines and regulations.

### 4.2. Lymphedema Mouse Tail Model

The current methods followed the previously published procedures [18]. Under general anesthesia, a 5 mm wide circumferential full-thickness skin section was excised 2 cm distal to the tail base to remove superficial L.V. After performing the incision of the operated area of skin, the location of L.V. was examined and the vein underneath the skin was peeled off the L.V. without inducing bleeding. Next, an examination was performed so that the injected Patent blue (116-11312 FUJIFILM Wako Pure Chemical Corporation, Osaka, Japan) did not show proximal leakage adjacent to the ligation and the L.V.s expanded at the operation site. The silicone splint (125–18–11-01, Tigers Polymer, Osaka, Japan) was sutured to the skin at both edges of the skin-peeled surgical field with 7–0 nylon (BEAR Medic Corporation, Tokyo, Japan), and the surgical procedure was completed.

### 4.3. Transdermal FITC Dextran and Steroid Administration Using Ultrasound Irradiation

**Figure 6:**
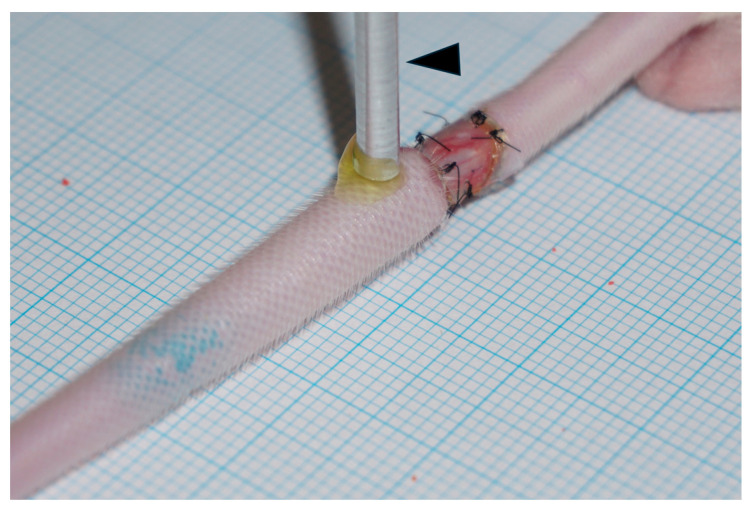
An image of ultrasound irradiation in progress. The black arrow indicates the ultrasound probe. FITC dextran or clobetasol propionate lotion was applied to the skin surface for the probe.

An ultrasound probe (Sonitron 2000N, Rich Mar, Inola, OK, USA) was utilized to deliver ultrasound directly to the tail skin through a 3 mm diameter probe. The duration time and other conditions were 10 min at an input frequency of 3.0 MHz, an output intensity of 3.0 W/cm^2^ (spatially averaged temporal peak; SATP), and a pulse duty cycle of 20%. During irradiation, 0.1 mL of clobetasol propionate lotion 0.05% (Rakool, Tokyo, Japan) or 2000 kDa of FITC dextran (PG research, Tokyo, Japan) was applied to the tail skin surface. In the edema model, the irradiated area was the edemic region adjacent to the surgical site (Figure 6). Ultrasound irradiation was performed at 7 days and 14 days after surgery.

### 4.4. Calculation of the Edema Status

**Figure 7:**
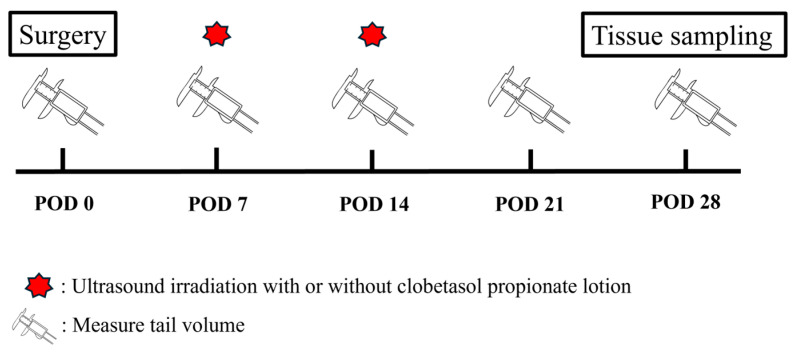
The timeline of the experimental procedure is shown in this figure. Ultrasonic irradiation was performed twice on POD7 and POD14. Tail measurements were performed every 7 days. The calculation of the tail volume was performed based on the equation by the measured width as shown in the methods. Tissue sampling was performed 28 days after surgical treatment (POD28).

The degree of edema formation was calculated weekly using the truncated cone formula which was reported previously: V = l/4π (C1C2 + C2C3… + C7C8) [18]. Tail diameters were measured using digital calipers (220-150-S, AS ONE CORPORATION, Osaka, Japan) every 0.5 cm, starting from the surgical site and going distally toward the tip of the tail. The timeline of the experiment is shown in Figure 7.

### 4.5. Histological and Fluorescent Immunohistochemistrical Assay

Tissue sampling was performed on POD 28 for histological examination. Lymphedema tail tissues were fixed in 4% paraformaldehyde at 4 °C for 24 h, decalcified using G-Chelate Mild (Geno Staff Co., Ltd., Tokyo, Japan), and embedded in paraffin. Paraffin sections were prepared at 6 μm and stained with hematoxylin and eosin (H&E) or Rabbit anti mouse CD4 antibodies (Cat# ab183685, EPR19514; Abcam, Cambridge, UK).

For immunofluorescent staining, antigen retrieval was achieved by Histo VT one (NACALAI TESQUE, Inc., Kyoto, Japan) at 105 °C for 10 min using an autoclave. Sections were incubated at 4 °C overnight with anti-mouse CD4 antibodies and incubated with secondary antibodies conjugated to Alexa Fluor 488 (A-11001, Thermo Fisher Scientific Waltham, MA, USA) for 1 h at room temperature. Sections were mounted with VECTASHIELD Vibrance Antifade Mounting Medium with DAPI (H-1800, Vector Laboratories Inc., Newark, CA, USA).

Frozen tissues were cryoprotected with 30% sucrose/PBS after decalcification, and embedded in OCT compound (Sakura Finetek, Tokyo, Japan) and 30% sucrose/PBS. Frozen sections of FITC dextran ultrasound irradiated tail were prepared at 10 µm. Frozen sections of tail lymphedema were prepared at 10 μm and stained by Oil red O stain stock solution (MUTO PURE CHEMICALS Co., Ltd., Tokyo, Japan). These sections were counterstained with hematoxylin and mounted with Glycergel mounting medium (109242 Sigma-Aldrich, St. Louis, MO, USA). Each section was imaged on a BZ-X800 fluorescence microscope (Keyence, Osaka, Japan). Cell counts for CD4-positive cells around a distance of 100 µm of lymphatic vessels were performed in each field (360 µm × 270 µm).

## 5. Conclusions

This study demonstrates the feasibility and effectiveness of using ultrasound irradiation for transdermal drug delivery in the treatment of secondary lymphedema. The ability to deliver large molecules and achieve significant therapeutic effects in an animal model is promising for the future development of noninvasive treatments. Continued research in this area could significantly improve the quality of life for patients suffering from secondary lymphedema by providing new, effective, and less invasive treatment options.

## Data Availability

The original contributions presented in the study are included in the article and further inquiries can be directed to the corresponding author/s.
